# Connecting the dots: Exploring bronchocentric granulomatosis in paediatric leukaemia

**DOI:** 10.1093/bjrcr/uaaf033

**Published:** 2025-07-07

**Authors:** Tanvi Pendse, Priscilla Joshi, Rucha Puranik, Divya Patel

**Affiliations:** Department of Radiodiagnosis, Bharati Vidyapeeth Medical College and Hospital, Pune, 411043, India; Department of Radiodiagnosis, Bharati Vidyapeeth Medical College and Hospital, Pune, 411043, India; Department of Radiodiagnosis, Bharati Vidyapeeth Medical College and Hospital, Pune, 411043, India; Department of Pathology, Poona Hospital, Pune, 411030, India

**Keywords:** chest, paediatric, rare

## Abstract

Bronchocentric granulomatosis is a rare form of granulomatous disease characterized by peribronchiolar or peribronchial necrotizing granulomas.^1^ The imaging findings are non-specific and include nodular or mass-like lesions and pneumonic consolidation.^2^ We present a case of bronchocentric granulomatosis in a patient with Pre-B-cell acute lymphoblastic leukaemia. The aim of the case report is to make the radiologist aware of this condition and emphasize the importance of multimodality approach which along with clinical findings helps in reaching a diagnosis and managing this rare complication.

## Introduction

Bronchocentric granulomatosis is a distinct histopathological entity characterized by necrotizing granulomas which are centred around bronchi and do not invade the pulmonary arteries. Bronchocentric granulomatosis is rare, although the exact incidence and prevalence are unknown.^3^ It is most commonly associated with connective tissue diseases, asthma, and allergic bronchopulmonary aspergillosis. An association with haematological malignancies, especially leukaemia, is extremely rare. We present a case of bronchocentric granulomatosis in a patient with acute lymphoblastic leukaemia.

## Case presentation

A 6-year-old male, known case of acute lymphoblastic leukaemia, presented to our hospital with a recurrent history of progressive dyspnoea, non-productive cough, and fever. He had attained remission and was on maintenance chemotherapy.

Physical examination revealed bilateral coarse crackles on lung auscultation. Laboratory investigations showed an elevated white blood cell count with a predominance of lymphoblasts. Serological tests for connective tissue diseases were negative.

He was referred to the Department of Radio-diagnosis at our hospital for multiple imaging modalities.

**Figure 1.**
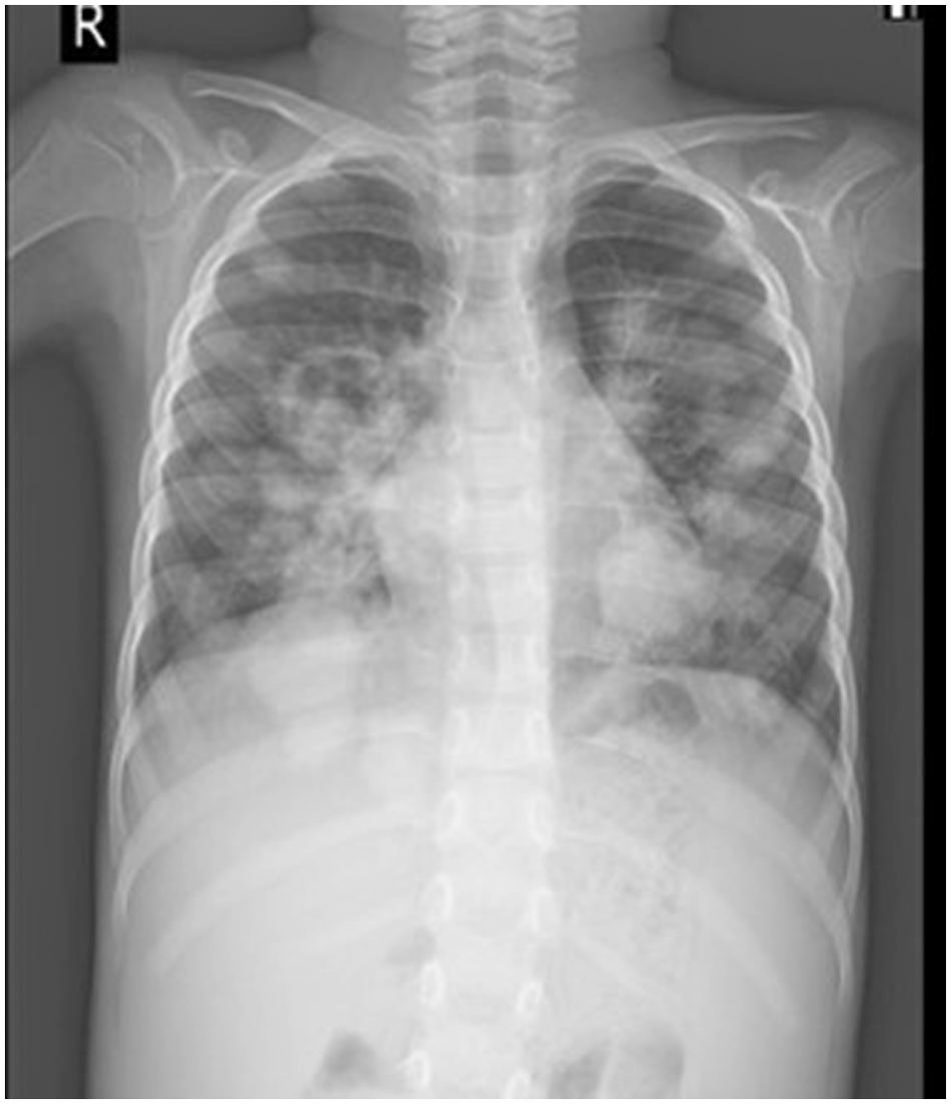
Chest X-ray showing right parahilar opacity with cavitation, opacity in the retrocardiac region, and radio-opacity silhouting the right diaphragm. After this serial, X-rays were done and CT in Figure 2 was done after 1 year due to repeated infections/admissions.

Chest X-ray revealed multiple ill-defined nodular opacities in bilateral lung fields. Multiple round opacities were seen in all the pulmonary lobes with cavitation in few nodules. A radio-opacity was noted in the retrocardiac region on the left and in the right lower zone, silhouetting the right cardiac border and right diaphragm (Figure 1).

**Figure 2.**
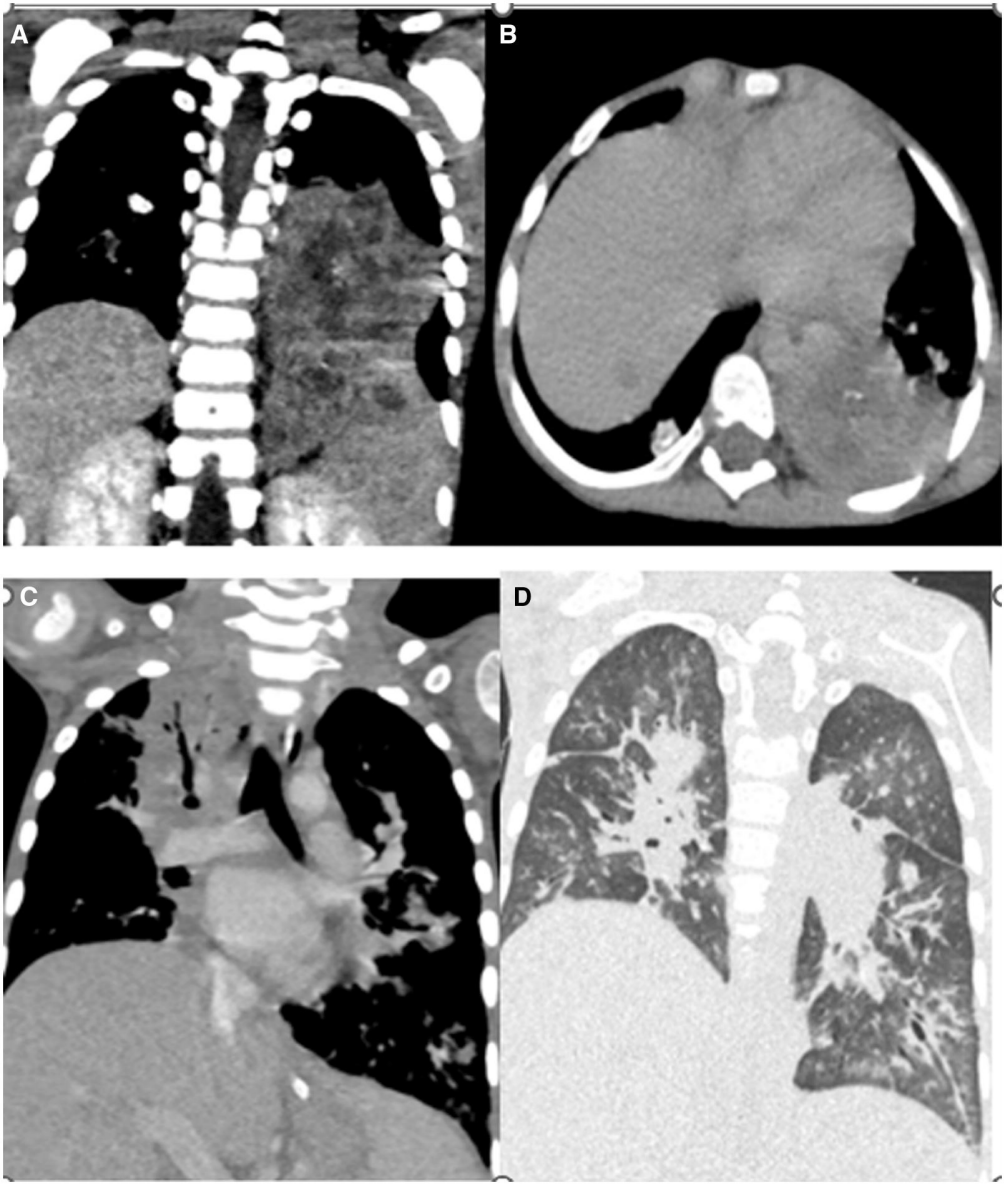
(A) Coronal and (B) axial post-contrast CT chest images showing a large heterogeneously enhancing parenchymal lesion in left lower lobe with areas of necrosis within causing complete occlusion of left lower lobe bronchus. (C) Coronal post contrast and (D) coronal CT chest images post corticosteroid therapy after 1 month showed complete resolution of left lower lobe lesion but showed increase in right perihilar infiltrates.

High Resolution Computed Tomography (HRCT) chest revealed multiple nodules in the upper and lower lobes bilaterally, right middle lobe, and lingula. Few of these showed calcification within with surrounding ground glass opacities. A large heterogeneously enhancing parenchymal lesion was seen in the left lower lobe with areas of necrosis within, causing complete occlusion of the left lower lobe bronchus. A lesion showing similar morphology was noted in the right perihilar region encasing the right upper lobe bronchus completely occluding the lumen (Figure 2).

**Figure 3.**
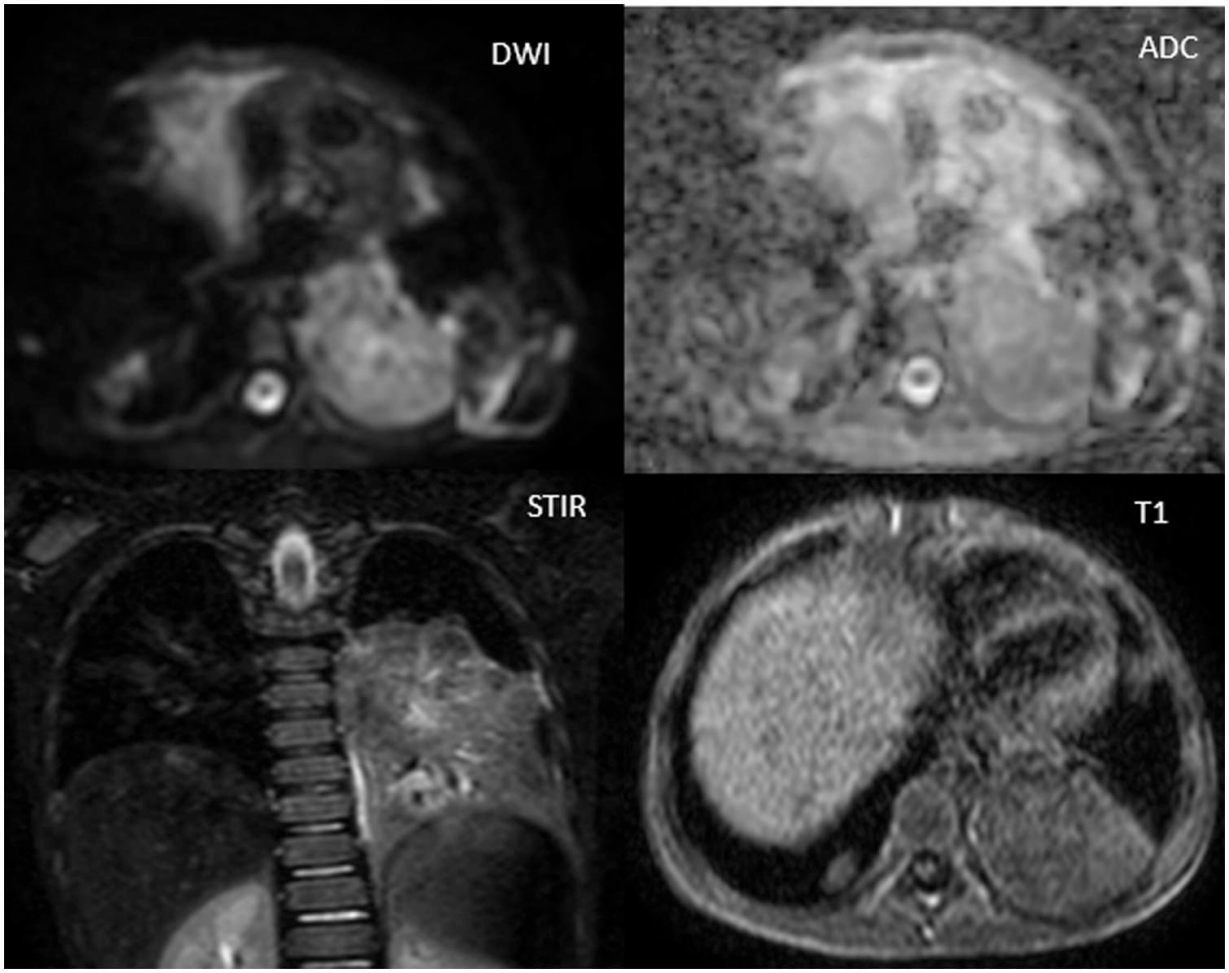
MRI thorax showing left parenchymal lesion appearing hypo-isointense on T1 and isointense on STIR images. No diffusion restriction in the mass.

MRI thorax showed no diffusion restriction in the mass ruling out high cellularity in the lesion. It also appeared hypo-isointense on T1 and isointense on STIR images (Figure 3).

**Figure 4.**
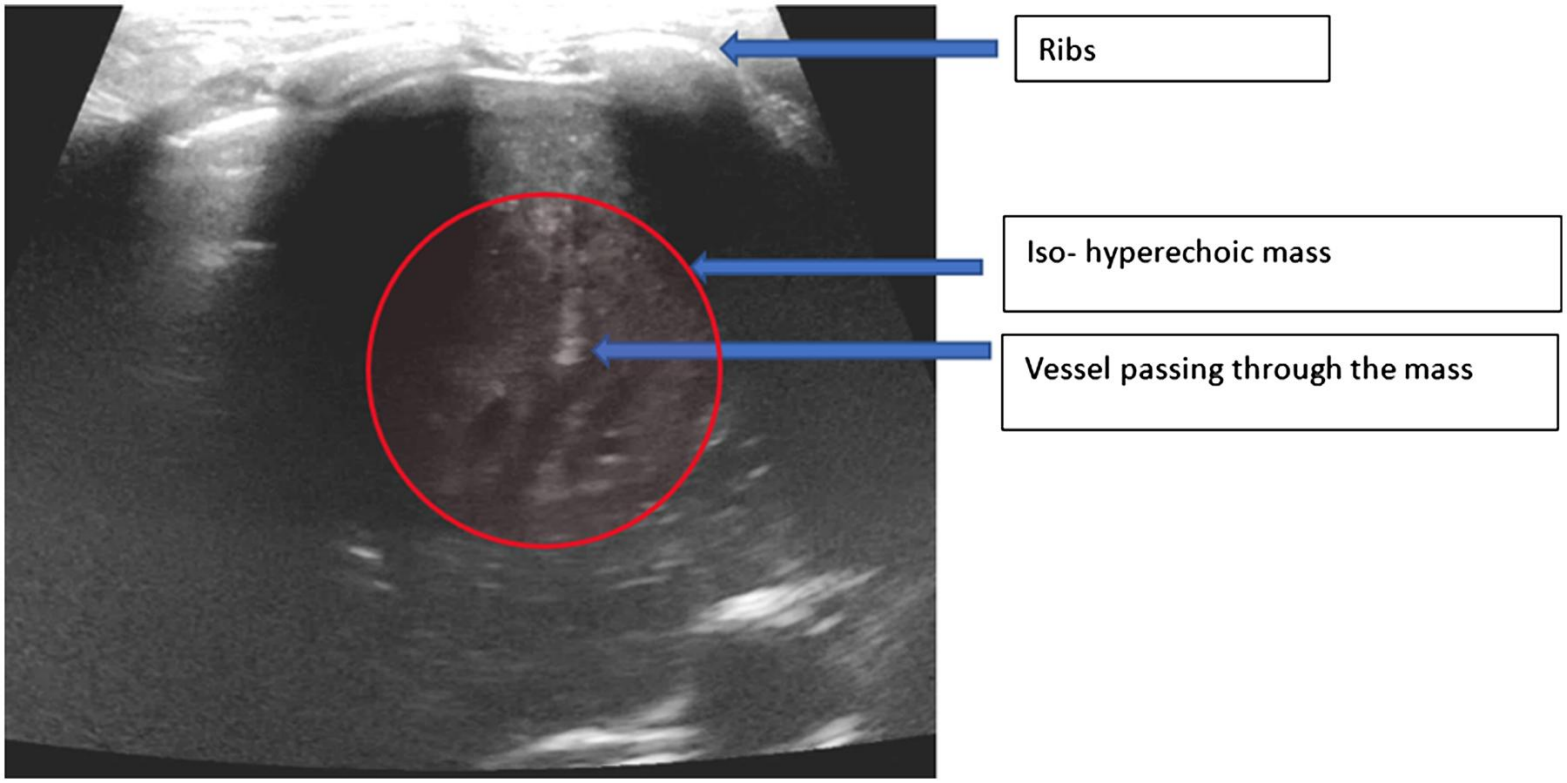
Academic USG revealed a iso-hyperechoic solid mass with vessels passing through it. No air bronchogram to suggest consolidation. Mass effect on left hemidiaphragm was noted.

Ultrasonography (USG) chest revealed an iso to hyperechoic solid mass in the left lower lobe with vessels traversing it causing mass effect on the left hemidiaphragm (Figure 4). No air bronchogram was seen within this hence ruling out a consolidation.

USG-guided biopsy of the left lower lobe lesion performed was negative for gram stain, AFB (Acid fast bacilli stain) KOH (potassium hydroxide), and calcofluor staining.

Bronchoscopy confirmed the CT findings and showed that the right upper and left lobe bronchus were covered with whitish fluffy deposits. The medial segment of right middle lobe, anterior segment of right lower lobe and left lower lobe bronchi were completely stenosed.

Culture of the bronchoalveolar lavage fluid showed an elevated cell count with a preponderance of lymphocytes and no fungal growth.

**Figure 5.**
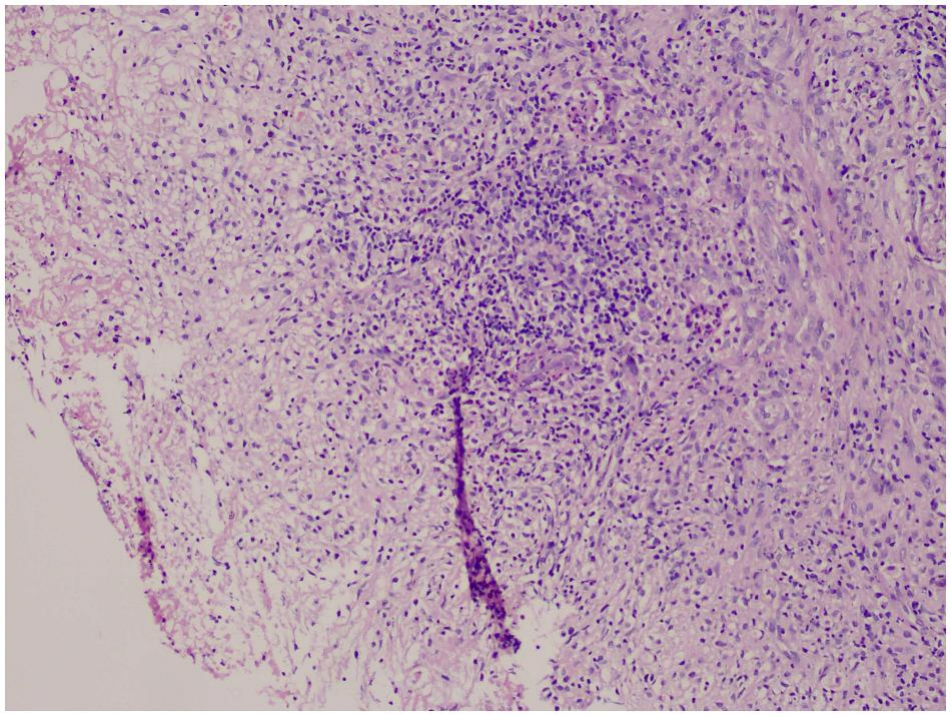
HPE suggestive of ulcerated and necrotic bronchial mucosa rimmed by epithelioid histiocytes with dense bronchial inflammation.

Histopathology of the endobronchial biopsy specimen revealed bronchocentric necrotizing granulomatous inflammation with increased eosinophils (Figure 5). No fungal elements were seen. Left lung lesion revealed chronic fibroinflammatory lesion with occasionally ill-defined granulomas, focal necrosis, and several eosinophils. Special stains for infectious agents (acid-fast bacilli, fungi) were negative. A bone marrow examination confirmed persistent leukaemia.

## Differential diagnosis

The left lower lobe lesion had multiple differential diagnosis like:

1. Neoplastic lesion—MRI showed no restricted diffusion thus ruling out a high cellularity mass lesion.
2. Consolidation was less likely due to the absence of air bronchogram.
3. Fungal infection—clinically, there was a high suspicion for fungal deposits but typical halo sign or multiple ground glass opacities were not seen. A mass-like lesion is usually not found in fungal infection.

The patient was finally diagnosed with bronchocentric granulomatosis in the setting of acute lymphoblastic leukaemia (ALL).

## Treatment and outcome

Corticosteroids were initiated for the bronchocentric granulomatosis along with IV antifungals as the fungal tissue culture grew scedosporium. The patient showed clinical deterioration with persistent high-grade fever, worsening wheeze, and respiratory distress requiring high-flow oxygen. Follow-up imaging revealed the left lower zone lesion had resolved after initiation of steroids. However, the right-sided upper zone infiltrates had worsened, the child slipped into sepsis with a poor outcome.

## Discussion

Bronchocentric granulomatosis is a rare histopathological entity typically associated with connective tissue diseases and fungal hypersensitivity but rarely linked to haematological malignancies. Granulomatous diseases show granulomas at imaging but have a wide variety of clinical manifestations that can mimic other conditions.

On CT, bronchocentric granulomatosis appears as a spiculated mass lesion or lobar consolidation with slight volume loss.^4^

Bronchocentric granulomatosis, although uncommon, should be suspected in patients with single or multiple lung nodules, mucoid impaction, or an area of consolidation that fails to respond to antibiotic therapy.^5^

In our case, the presence of bronchocentric granulomatosis in a patient with ALL posed a diagnostic challenge due to its atypical association. In our case, careful evaluation of clinical presentation, imaging findings, and histopathology were crucial in arriving at the right diagnosis and managing the condition.

## Conclusion

Our case highlights the importance of unusual manifestations of pulmonary diseases in patients with haematological malignancies.

Multimodality radiological techniques along with discussion with haematologists, pulmonologists, and pathologists will aid in timely diagnosis and proper management of such complex case. This condition warrants a high level of suspicion, given its rarity, especially in cases with poor response to treatment.

## Learning points

- Differential of a neoplastic lesion was ruled out as MRI did not show any diffusion restriction and CT did not show any invasion in surrounding structures.
- Consolidation was also suspected initially but USG and CT did not show any air bronchogram thus ruling out the possibility.
- As typical findings expected in fungal infections were also not seen, our suspicion went towards a rare pathology.
- Bronchocentric granulomatosis is usually characterized by necrotizing granulomas which do not invade the pulmonary arteries which is similar in our case. Hence other conditions like granulomatosis with polyangiitis, necrotizing sarcoidosis, and lymphomatoid granulomatosis were ruled out as they have tendency to involve arteries.
- As this is histopathologically proven diagnosis, consultation with pathologists is important for timely diagnosis.
- Prognosis of bronchocentric granulomatosis varies on the severity of the disease and the presence of other underlying conditions.
